# 

**Published:** 1910-03-05

**Authors:** 


### Appointments.

Dr. J. P. Grieves has been appointed medical officer of health to the Portishead Urban District Council.

Dr. C. H. Adamson, F.R.C.S., has been appointed honorary medical consulting officer to the Royal Victoria Hospital, Dover.

Dr. W. G. Ellis has been appointed principal civil medical officer of the Straits Settlements, in place of Dr. D. K. McDowell, C.M.G.

Mr. Arnold Lawson, F.R.C.S.Eng., L.R.C.P.Lond., M.D.(Brux.), has been appointed assistant ophthalmic surgeon at the Middlesex Hospital.

Mr. George Herbert Colt, M.A., M.B., B.C.Cantab., F.R.C.S., L.R.C.P.London, has been appointed an assistant surgeon at Aberdeen Royal Infirmary.

Dr. H. J. Gibbs, medical officer of the Tan Tock Sengs Hospital, Singapore, has been selected for the office of medical superintendent of the Lunatic Asylum, Singapore.

Mr. H. C. T. Langdon has been appointed medical officer of health for Mansfield, medical officer of the local infectious diseases hospital, and medical examiner of schools.

Dr. McDowell, after nearly six years of service in Singapore as principal civil medical officer, has been transferred to the Federated Malay State as chief of the Medical Department. Before proceeding to the East in 1903 he served in the West Indies and on the West Coast of Africa, and was attached to the Ashanti Expeditionary Force in the year 1900 as principal medical officer.

The following gentlemen have been selected as house officers at St. Thomas's Hospital *from Tuesday, March 1*, 1910:—*Casualty Officers and Resident Anæsthetists.*—G. B. Wainwright, L. S. T. Burrell, R. Cox, L. B. Perry, L. Meakin, G. Hoffmann. *Casualty Assistants.*—E. M. Lauderdale, E. A. Seymour. *Resident House Physicians* (*from February* 15, 1910).—H. W. C. Topley, H. P. Newsholme, F. C. Pridham, H. Bowring, M. E. Dellschaft. *Resident House Surgeons* (*from February* 15, 1910).—B. C. Maybury, A. F. Morcom, J. S. Hopwood, W. R. Parkinson. *House Surgeon to Block* 8.—W. B. Johnson. *Obstetric House Physicians.*—(Senr.) J. L. Graham-Jones, (Junr.) M. W. Baker. *Ophthalmic House Surgeon.*—(Senr.) T. C. R. Archer, (Junr.) W. Victor Corbett. *Clinical Assistants.*—Throat: (Senr.) W. G. Howarth, W. L. Pink; Skin: W. N. Child; Ear: (Senr.) W. G. Howarth, W. L. Pink; Children's Medical: L. M. Routh, C. Newcomb; Mental: W. Victor Corbett.

### Gifts and Bequests.

Mr. R. Nivison has given £1,000 towards the special appeal fund of St. Bartholomew's Hospital.

Mrs. Morrison has left £5,000 to the Anti-Vivisection Hospital, Battersea Park Road, S.W.

Mrs. Eliza Wyatt, of Upper Westbourne Terrace, has left £100 to St. Mary's Hospital, Paddington.

Miss Alice Leontine Hannah Goetz, of Lower Seymour Street, S.W., has left £100 to the London Hospital.

Mr. Thomas Nussey, retired woollen merchant, formerly of the firm of Messrs. Hargreaves and Nussey, of Wortley, near Leeds, left £200 to the Leeds Infirmary and £100 to the Leeds Dispensary.

Mr. Isaac Falcke, a well-known art collector, has left £50 to the Jews' Hospital and Orphan Asylum. On the death of his wife he left £100 to University College Hospital and £50 to the Middlesex Hospital.

Mr. Donald Cameron, of Stirlingshire, and formerly Mayor of Windsor, Ontario, has left £1,000 to the Belford Hospital, Fort William, £500 each to the Royal Infirmary, Glasgow, and the Western Infirmary, Glasgow.

Mrs. Elizabeth Cooper, of Denmark Hill, S.E., has left £1,000 each to Guy's Hospital, the Royal Dental Hospital, Charing Cross Hospital, the London Hospital, and the British Home for Incurables at Streatham.

Mr. Henry James Leggett, of London, has left the residue of his estate (estimated at over £6,000) equally between the Great Northern Central Hospital, the Royal Free Hospital, Gray's Inn Road, and Southgate Hospital, and one other non-medical institute.

Among the latest contributions received for King Edward's Hospital Fund for London are the following: Annual subscriptions—Bank of England, £250; Sir John Aird, £105; Messrs. John Aird and Sons, £105; Sir Thomas de la Rue and Co., £52 10s.; Sir William James Farrer, £50; donation—Mr. Robert Nivison, £1,000.

Mr. Richard Laybourne, J.P., D.L., of The Firs, Malpas, Monmouth, civil engineer, and of the Isca Foundry Company, left estate valued at £103,022 gross, with net personalty £93,387. He bequeathed £1,000 ordinary stock of the Newport (Mon.) Gas Co. to the Newport and Monmouthshire Hospital, for the endowment of a bed in the Women's Medical Ward.

Mr. George Henry Swonnell, of Wandsworth Common, S.W., has left £500 to the Bolingbroke Hospital, and £250 each to the Norwood Cottage Hospital and the Croydon General Hospital. The residue of his estate, which will probably amount to £27,000 (which, however, is subject to a deduction of about £2,700 for legacy duty), the testator left to King Edward's Hospital Fund for London.

Miss Emma Brandreth, of Wimbledon Park, S.W., has left £1,000 to King Edward's Hospital Fund for London; £500 to the Royal London Ophthalmic Hospital; £250 each to Guy's Hospital, the Middlesex Hospital, the East London Hospital for Children, Shadwell, the Poplar Hospital for Accidents, the Royal Sea Bathing Hospital, Margate, the Cheyne Hospital for Sick and Incurable Children, the Royal Dental Hospital, London, the London Fever Hospital, the New Hospital for Women, Euston, St. Peter's Hospital for Stone, the Royal Free Hospital, Gray's Inn Road, Church Army, the Bolingbroke Hospital, Wandsworth. The residue of her estate, which will probably amount to about £29,000 (subject to a deduction of £2,900 for legacy duty), Miss Brandreth left to the executors to distribute among deserving and needy charitable hospitals or institutions.

### THE MEDICAL LIBRARY.

| | Post Free. |
|---|---|
| "Medical History: From the Earliest Times." By E. T. Withington, M.A., M.B. Oxon ... | 12s. 6d. |
| "The Consumptive Working Man. What Can the Sanatoria Do for Him?" By Noel D. Bardswell, M.D. ... ... ... ... ... ... | 10s. 10d. |
| "Photomicrography." By Edmund Spitta, M.R.C.S. With 41 Half-tone Reproductions from original negatives and other Illustrations ... ... | 12s. 0d. |
| "Outlines of Insanity." A Popular Treatise on the Salient Features of Insanity. ... ... ... | 1s. 6d. |
| "Sight and Hearing in Childhood." By R. Brudenell Carter, F.R.C.S., and Arthur Cheatle, F.R.C.S. | 2s. 3d. |

Of all booksellers or of The Scientific Press, Limited, 28 & 29 Southampton Street, Strand, London, W.C.

### THE QUESTION BOX.

**SPECIAL NOTICE.**—Questions of interest affecting the honorary medical staff and hospital administration in all departments are invited. When any point arises we hope our readers will formulate it in a question and so co-operate to make this column of real value and interest. In this way the experience of the best-managed hospitals should be made helpful to the whole hospital world. We shall welcome the contribution of both questions and answers.

N.B.—The publication of an answer in this column does not necessarily preclude the publication of subsequent answers to the same question, for a question may involve points which require to be threshed out and fully discussed. Answers, if published, will be paid for at scale rates.

### A CENTRAL STORE FOR HOSPITALS.

### A Metropolitan View.

By a Hospital Officer.

The suggestion of a Central Store for Hospitals is not likely to be a practicable one, for the reason that the expense of the establishment and upkeep of such a central store would be very great, and hardly likely to be outweighed by the advantages to be derived from its establishment. The institutions benefiting by it, to any appreciable extent, would probably only be the smaller hospitals, which would certainly gain the advantage of buying their comparatively small stock of requirements at the same prices as any of the large hospitals.

If it is established that an alteration is necessary or desirable in the present arrangement of each hospital catering for its own needs, I think it should be rather in the direction of combination of contracts, tenders being invited, and contracts made by some central authority, such as King Edward's Hospital Fund. This central authority would ascertain from the various hospitals their approximate requirements for the stated period of contract, and make contracts for the total requirements, each hospital then sending their orders direct to the contractors, as they do at present, thus retaining direct communication with, and control over them. This arrangement, however, although obviating the establishment of an expensive central store, and the consequent outlay of a large amount of capital, would similarly mainly benefit the smaller hospitals, which cannot under present conditions make such advantageous catering arrangements as the larger institutions, and it would probably be found "that in practice a well organised commissariat department at a great hospital can purchase the best articles in requisite quantities at a price so low as to render the institution of one Central Hospital Store unnecessary, or even undesirable.

### A Manchester View.

By Mr. W. G. Carnt, General Superintendent and Secretary, Manchester Royal Infirmary.

I do not think a central store for hospitals a feasible proposition. This is a subject which has cropped up periodically for many years. Hospital administrators have discussed it, have decided that it would be an excellent arrangement if it could be worked economically, and have finally dismissed it, as the difficulties in the way of establishing a scheme of this nature have appeared insuperable, but it is such a fascinating subject that it will continue to recur from time to time, and I have no doubt that the conclusion on each future occasion will be the same as it has been hitherto. The equipment of a modern hospital is so complex, hospital administrators differ so widely upon points of taste in furniture, etc., both for the hospital administrative blocks and for the wards, doctors differ so considerably upon the question of hospital necessaries, that standard articles for use in any group of hospitals is a practical impossibility, and without standard patterns I cannot see that a central store for hospitals is likely to obtain a sufficient reduction upon the cost of supplies to justify its existence. The cost of providing and stocking such a store would be enormous, and the capital necessarily lying idle, even if it could be provided, would be a serious item which would have to be considered in fixing the prices of the articles. Again, to make such a store a success, men of wide experience in the variety of goods dealt in would have to be engaged at salaries sufficiently tempting to ensure the continuance of their services. Their salaries would have to be recovered from the purchasing institutions through the medium of the charge for the goods supplied.

The idea of a central store is attractive, but when the details are thought out and hard facts are considered it assumes an impracticable appearance.

Although a central store appears to be impossible, there does seem to be a possible combination of hospitals which could ensure the purchase of a great many supplies in a cheaper market than individual hospitals could deal in. If a number of institutions would combine and appoint representatives to form a Contract Committee authorised to invite tenders for the supply of certain necessaries to the whole of those hospitals, the selected firm or firms to deliver goods contracted for to the order of the individual hospitals, lower prices might be obtained, which would result in the aggregate in a considerable saving. The goods which could be contracted for under this arrangement would be the following :—(1) All provisions. (2) Drugs. (3) A considerable proportion of the dressings. (4) Wines and spirits. (5) Bottles and many dispensary sundries. (6) Certain cotton and linen goods. (7) Coal. (8) Soap, soda, brushes and cleaning articles, and some other items. There would be, of course, in all the hospitals in the group a number of special requirements which would have to be obtained apart from the fixed contracts, but this is unavoidable, and these exceptions need not be taken into consideration, as, if no combination existed, they would probably not be included by any hospital in contracts covering a period. This scheme could, of course, only be worked in large centres where hospitals are numerous and transit easy. It would be impossible for the great bulk of the hospitals in the country to take part in any such combination.

The scheme outlined would cost practically nothing in administration beyond printing and postage, as, when once the contracts were fixed for a period, the labours of the committee would end until contract time again arrived.

---

The committee of the Royal Victoria Hospital, Bournemouth, has received a legacy of £500, less estate duty, from the executors of the late Miss Duer, on condition that a bed be named after the testatrix and that the institution take care of her grave in perpetuity.

During the past year the Bristol Royal Infirmary has received the following legacies: Robert Burges Sayer (balance), £35 11s. 11d.; James L. Perrin, £500; John Wyatt, £89 15s.; G. A. Gerrish, £100; Miss Frances Burges Brown, £177 15s. 7d.; Col. E. Podmore Clark, £25; Miss Adelaide Hartley, £100; Miss Eliza Young, £50; Charles Hollwey, £105 15s. 3d.; Miss Sarah Moss, £5; Arthur Baker, £100; Miss Fanny Case, £1,527 17s. 8d.; Edward Bush, £600; Robert Townsend Hippisley, £100; Miss Emily Lucas, £50.

THE
### GRADUATES' MEDICAL SCHOOLS.

DIARY FOR THE WEEK, MARCH 7 to MARCH 12.

**MEDICAL SOCIETY OF LONDON, Chandos Street, Cavendish Square, W.**

**At 9 p.m.—March 7, Lettsomian Lecture III.,** Dr. J. S. Risien Russell, **The Cerebellum and its Affections.**

**LONDON SCHOOL OF CLINICAL MEDICINE, Seamen's Hospital, Greenwich, S.E.**

**At 2.15 p.m.—March 7,** Mr. W. Turner, **Stricture.**
**2.15 p.m.—March 8,** Professor Hewlett, **The Wassermann Reaction.**
**3.15 p.m.—March 11,** Mr. McGavin, **The Surgery of the Thorax.**

**THE BROMPTON HOSPITAL FOR CONSUMPTION AND DISEASES OF THE CHEST, Fulham Road, S.W.**

**At 4 p.m.—March 9,** Dr. Theodore Williams, **The Relation of Fibrosis to Tuberculosis.**

**NORTH-EAST LONDON POST-GRADUATE COLLEGE, Prince of Wales's General Hospital, Tottenham, N.**

**At 4.30.—March 8,** Mr. J. H. Evans, **Some Points in the Surgery of the Aged.**
**March 10.**—Dr. G. B. Price, **The Clinical Features of Some Unusual Abdominal Tumours.**

**MEDICAL GRADUATES' COLLEGE AND POLYCLINIC, 22 Chenies Street, W.C.**

**At 4 p.m.—March 7,** Dr. H. G. Adamson, **Clinique (skin).**
**5.15 p.m.—March 7,** Dr. F. J. McCann, **The Treatment of Puerperal Infection.**
**4 p.m.—March 8,** Sir William Gowers, **Clinique (medical).**
**5.15 p.m.—March 8,** Mr. Nathan Raw (Liverpool), **The Treatment of Pulmonary Tuberculosis by Bovine Tuberculin.** (Results of Treatment.)
**4 p.m.—March 9,** Mr. Mower White, **Clinique (surgical).**
**5.15 p.m.—March 9,** Mr. G. Lenthal Cheatle, **Cancer of the Tongue.**
**4 p.m.—March 10,** Mr. Jackson Clarke, **Clinique (surgical).**
**5.15 p.m.—March 10,** Dr. A. P. Luff, **The Treatment of Some of the Forms of Chronic Rheumatism.**
**4 p.m.—March 11,** Dr. J. Dundas Grant, **Clinique (ear, nose, and throat).**

**CENTRAL LONDON THROAT AND EAR HOSPITAL, Gray's Inn Road, W.C.**

**At 3.45 p.m.—March 8,** Dr. Andrew Wylie, **Larynx.**
**4.30 p.m.—March 10,** Dr. Wyatt Wingrave, **A Summary of Recent Pathological Experience in the Practice of the Hospital.**
**3.45 p.m.—March 11,** Dr. Andrew Wylie, **Larynx.**

**THE POST-GRADUATE COLLEGE, West London Hospital, Hammersmith, W.**

**At 10 a.m.—March 7 and 10, Demonstration by Surgical Registrar.**
**10 a.m.—March 11, Demonstration by Medical Registrar.**
**12 noon.—March 10,** Dr. Bernstein, **Pathological Demonstration.**
**12.15 p.m.—March 9,** Dr. Grainger Stewart, **Practical Medicine.**
**5 p.m.—March 7,** Mr. Dunn, **Some Clinical Facts in the Study of Ophthalmic Disease.**
**5 p.m.—March 8,** Mr. Pardoe, **Clinical Lecture.**
**5 p.m.—March 9,** Dr. Beddard, **Medicine.**
**5 p.m.—March 10,** Dr. Davis, **Tonsillitis, Quinsy, Diphtheria, and Treatment.**
**5 p.m.—March 11,** Dr. Grainger Stewart, **Compression Paraplegia.**

**ROYAL SOCIETY OF MEDICINE, 20 Hanover Square, W.**

**At 5.30 p.m.—March 8, Surgical Section:**
*Papers.*—Mr. Somerville Hastings: "Rupture of the tunica vaginalis in hydroceles." Dr. W. Soltau Fenwick: "The clinical significance of gastric hypersecretion, and its connection with latent disease of the appendix." Mr. H. Paterson: "Appendicular gastralgia or the appendix as a cause of gastric symptoms."

**5.30 p.m.—March 9, Balneological and Climatological Section:**
*Discussion* on "Arthritis deformans" will be opened by Dr. Llewelyn Jones (Bath). Members of the Medical and Therapeutical and Pharmacological Sections are specially invited to attend this meeting.

**7.45 p.m.—March 10, Obstetrical and Gynæcological Section:**
*Specimens.*—Dr. F. J. McCann: (1) Sarcoma of cervix removed by abdominal colpo-hysterectomy. (2) Abdominal pregnancy.
*Demonstration with epidiascope.*—Mr. T. Carwardine: Microscopical appearances in tubal pregnancy.
*Short communication.*—Dr. Russell Andrews: Carcinoma of the cervix of a prolapsed uterus with contact carcinoma of the vulva.

**5 p.m.—March 11, General Meeting of Fellows: Election of Candidates for Fellowship.**

**8 p.m.—March 11, Clinical Section:**
*Clinical cases.*—Mr. Pearce Gould: Detachment of entire scalp treated by grafting. Mr. Maynard Smith: Obliterative endo-aneurysmorrhaphy (Matas's operation) for popliteal aneurysm. Mr. C. Gordon Watson: Tibio-calcaneal resection with amputation of the rest of the foot. Mr. Carless: Lymphangioma of the tongue. Mr. J. Howell Evans: X-ray carcinoma of the hand, illustrated by specimens. Dr. Galloway: (1) Spleno-medullary leukæmia with intercurrent erysipelas. (2) Trophic dermatitis of extremities.

